# Complete Genome Sequence of the Methicillin-Resistant Staphylococcus aureus Strain SQL1/USA300, Used for Testing the Antimicrobial Properties of Clay Phyllosilicates and Customized Aluminosilicates

**DOI:** 10.1128/MRA.00861-21

**Published:** 2021-11-11

**Authors:** Enrique G. Medrano, Shelley E. Haydel

**Affiliations:** a Insect Control and Cotton Disease Research Unit, U.S. Department of Agriculture, Agricultural Research Service, College Station, Texas, USA; b School of Life Sciences, Arizona State University, Tempe, Arizona, USA; c Biodesign Institute Center for Bioelectronics and Biosensors, Arizona State University, Tempe, Arizona, USA; University of Rochester School of Medicine and Dentistry

## Abstract

Methicillin-resistant Staphylococcus aureus (MRSA) is a Gram-positive bacterium that causes community-acquired and health care-acquired infections. We previously demonstrated that clay phyllosilicates and customized aluminosilicates display antimicrobial activity against the MRSA strain SQL1. The SQL1 annotated genome reveals a USA300 lineage and contributes critical knowledge of the MRSA virulence factors associated with tissue infection.

## ANNOUNCEMENT

Methicillin-resistant Staphylococcus aureus (MRSA) infections are increasingly more difficult to treat due to broad antibiotic resistance (1–5). In continuity, our studies have incorporated the same MRSA strain (provided as a gift from Sonora Quest Laboratories, Tempe, AZ, USA [6]) to examine the utility of clay constituents as antimicrobials (6–9). The MRSA strain (MRSA SQL1) was sequenced to provide genetic insights into both the biology and infection paradigm employed by the disease agent.

MRSA SQL1 was grown in Trypticase soy broth (Becton-Dickson, Sparks, MD, USA) at 37°C for 16 to 20 h. Plasmid DNA and genomic DNA were isolated using a standard plasmid miniprep kit and genomic DNA isolation procedure, respectively, with lysostaphin and proteinase K added to the lysis buffers, and purified using silica spin columns (QIAprep miniprep kit and genomic DNA purification kit, respectively; Qiagen, Valencia, CA, USA). The strategy used to sequence and annotate the genome of MRSA SQL1 essentially followed that of Medrano et al. (10). A Pacific Biosciences Sequel instrument was used to perform sequencing with the SMRTbell Express template preparation kit v2.0 with >10-kb fragments. DNA shearing with a Covaris g-TUBE assembly (Woburn, MA, USA) preceded size selection on a BluePippin system (Sage Science, Beverly, MA, USA) following manufacturer protocols. The library was sequenced using a 10-h movie collection time with a single-molecule real-time (SMRT) cell 1M v8, producing 230,419 reads with a 2.1-Gb molecular yield and a mean subread length of 8.9 kb (*N*_50_, 9.7 kb). The genome was assembled using SMRT Link v9.0.0.92188, with the Microbial Assembly protocol standard settings, including the read quality control, error correction, and adapter trimming functions, were employed, with an expected genome size setting of 2 Mb. The genome completeness was based on minimap2 v2.17 using standard PacBio recommended coding (i.e., ax map-pb) that mapped the reads back to the circularized genome and verified the reads that spanned the junction (11). The Microbial Assembly application of SMRT Link performs circularization and trimming and rotates the assembly to place the origin of replication at the beginning of the final linearized assembly. The finalized assembly included three circular contigs with a chromosome of 2.9 Mb (GC content, 32.7%) and extrachromosomal plasmids of 27.1 kb (GC content, 30.5%) and 3.1 kb (GC content, 28.7%); the three contigs had approximately 520×, 864×, and 439× coverages, respectively. The Prokaryotic Genome Annotation Pipeline program v4.11 at the NCBI was used to computationally annotate the sequence data (12).

**FIG 1:**
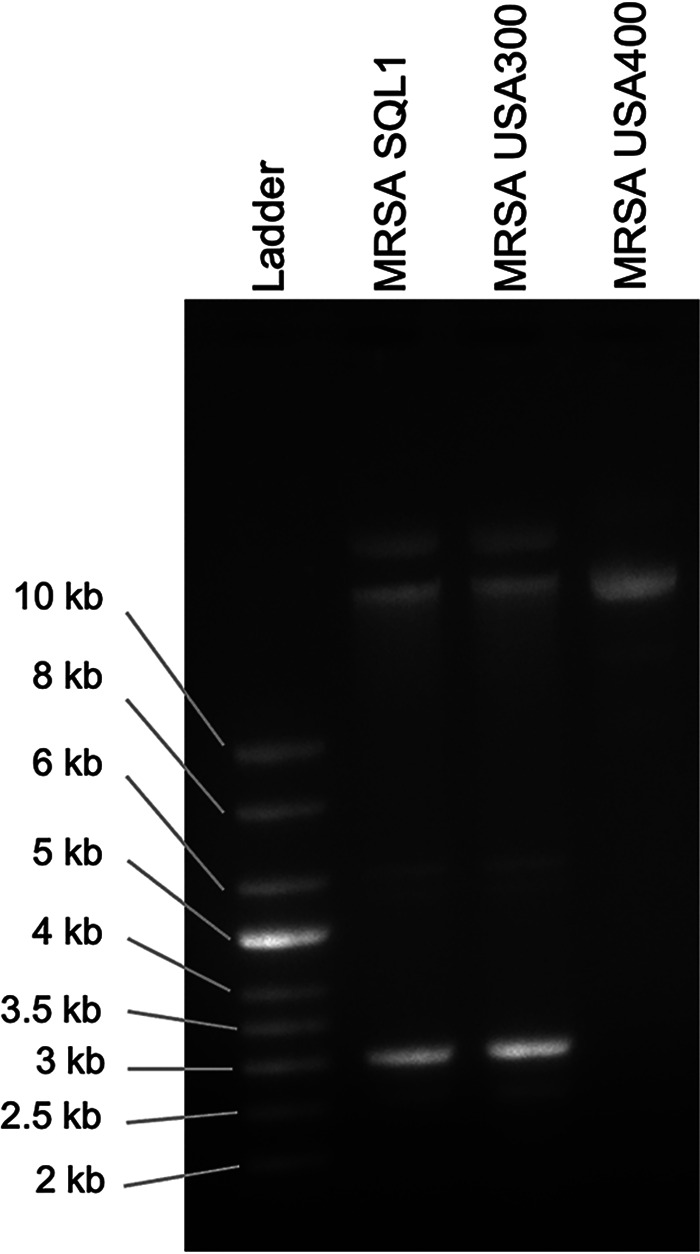
Plasmid profiles of MRSA strains SQL1, USA300, and USA400. Plasmids were isolated from the three MRSA strains and separated on a 0.5% agarose gel. The MRSA SQL1 plasmid profile is identical to the known MRSA strain USA300.

From a total of 2,919 computed genes, 2,837 had predicted coding DNA sequences for the genome, with 19 rRNA operons and 59 tRNAs. The annotation data identified a putative *mecR1* fragment and the Pantón-Valentine leukocidins *lukS* and *lukF*, all evidence of a USA300 lineage. An extrachromosomal DNA profile provided further evidence of similarity between SQL1 and USA300 (Fig. 1), correlating with sequencing results revealing plasmids of 27.1 kb and 3.1 kb. Additional virulence determinants included open reading frames for the *luxA*/*C* siderophore, lysostaphin protein A, and *msrA* macrolide efflux pump genes. Generally, the strain SQL1/USA300 data presented advance our ongoing development of novel MRSA infection treatment strategies (13–16).

### Data availability.

The assembled whole-genome sequences were deposited at DDBJ/EMBL/GenBank (accession numbers CP081354.1, CP081355.1, and CP081356.1). The raw data are available in the SRA database (accession number SRX11799045) with general details available under BioProject accession number PRJNA751845.
